# A multimodal multitask deep learning framework for vibrotactile feedback and sound rendering

**DOI:** 10.1038/s41598-024-64376-y

**Published:** 2024-06-10

**Authors:** Joolekha Bibi Joolee, Md Azher Uddin

**Affiliations:** Mathematical and Computer Sciences department, Heriot-Watt University Dubai, Dubai, 501745 United Arab Emirates

**Keywords:** Computer science, Information technology

## Abstract

Data-driven approaches are often utilized to model and generate vibrotactile feedback and sounds for rigid stylus-based interaction. Nevertheless, in prior research, these two modalities were typically addressed separately due to challenges related to synchronization and design complexity. To this end, we introduce a novel multimodal multitask deep learning framework. In this paper, we developed a comprehensive end-to-end data-driven system that encompasses the capture of contact acceleration signals and sound data from various texture surfaces. This framework introduces novel encoder-decoder networks for modeling and rendering vibrotactile feedback through an actuator while routing sound to headphones. The proposed encoder-decoder networks incorporate stacked transformers with convolutional layers to capture both local variability and overall trends within the data. To the best of our knowledge, this is the first attempt to apply transformer-based data-driven approach for modeling and rendering of vibrotactile signals as well as sounds during tool-surface interactions. In numerical evaluations, the proposed framework demonstrates a lower RMS error compared to state-of-the-art models for both vibrotactile signals and sound data. Additionally, subjective similarity evaluation also confirm the superiority of proposed method over state-of-the-art.

## Introduction

Human interaction with textured surfaces relies on the use of multiple senses, primarily vision, touch, and hearing. These senses play a vital role in perceiving objects during physical interactions. Due to the recent breakthroughs on computer graphics research, the transition from traditional 2D visual content to adaptive 3D mixed reality worlds is straightforward and showing promising results^1^. In haptics, most of the existing works for vibrotactile feedback rendering or sound rendering use a tool-based texture interaction approach, where the user moves the tool over the virtual texture, and vibrotactile feedback is rendered via an actuator^2–4^, or sound is rendered through headphones^5^. Independently, both vibrotactile feedback rendering and sound rendering demonstrated sufficient reconstruction accuracy when applying the data-driven paradigm. Simultaneously rendering both vibrotactile feedback and sound in a simulated environment presents significant challenges, particularly in terms of synchronization and design complexity^1^.

The initial research in the field of vibrotactile signal and sound modeling mostly concentrated on developing simulations using a physics-based approach. For instance, Gaussian-distributed force fields are employed to generate stochastic vibrotactile signal in^6,7^. McDonald et al.^8^ utilized a lumped-parameter model, an offset surface, and Hertzian contact mechanics for the dynamic simulation of tool-mediated texture interaction. Similarly, time and frequency domain linear prediction coding (TFLPC) was introduced to synthesize the sound of textures in^9^. Zheng et al.^10^ proposed a frictional multibody contact formulation to render sound. A parameter estimation algorithm is presented for sound synthesis in^12^, which overcomes the limitation of linear modal synthesis (i.e., frictional multibody contact formulation^10^). However, the introduction of more sophisticated textures (i.e., anisotropic textures) has led to challenges with the intricate physical models and computational demands in real-time physics-based simulations of tool-surface interactions^5,17,18^. Although simplification attempts have been made but at the cost of reducing perceptual realism, impacting virtual experience authenticity. Some earlier studies adopted record-and-playback method to render vibration signal^13,14^ and sound^15^.

In contrast, data-driven approaches offer a dynamic and effective means of modeling and generating complicated vibrotactile signals and sounds. Moreover, data-driven approach eliminates the need for intricate parameter adjustments and instead aim to establish correlations between user inputs (such as speed, force, and direction) and outputs (including vibrotactile signals and sound). In the early data-driven approach, vibrotactile signals were modeled and rendered using techniques such as Linear Predictive Coding (LPC)^16^, Auto Regressive Moving Average (ARMA)^2^, radial basis function network (RBFN)^17,18^, neural networks (NNs)^19^ and Waveform Segment Table^3^. Yet, despite these efforts, their performance was not adequate. Recently proposed deep learning-based data-driven approaches, i.e., the deep spatiotemporal network (DSTN)^4^ and the deep convolutional generative adversarial network (DCGAN)^20^, have exhibited substantial improvements over previous methods for vibrotactile signal modeling and rendering. Additionally, data-driven modeling based on Wavelet trees is employed for rendering the sounds of tool-surface interactions^5^.

While numerous approaches have been introduced to render vibrotactile feedback and sound independently, limited effort has been directed towards their simultaneous rendering. For instance, Sterling et al.^21^ exhibited that normal maps and relief maps can serve as integrated representations of intricate surface details and render haptic textures as well as synthesize sounds. In^1^, object geometry and material properties, including displacement and roughness texture maps, are employed within a physics-based model to achieve the realistic rendering of both vibrotactile feedback and sound. However, these existing approaches rely on physics-based modeling and may encounter issues inherent to such modeling techniques.

Our hypothesis is that the utilization of emerging deep-learning methodologies featuring multitask learning has the potential to enhance the performance of rendering vibrotactile feedback and sound simultaneously. This is further encouraged by recent works that involve deep-learning models with multitask learning in diverse tasks, e.g., Alzheimer’s disease progression detection^22^, financial risk forecasting^23^, and face pose estimation^24^.

Inspired by the aforementioned studies, in this paper we present a multimodal multitask deep learning framework for the simultaneous modeling and rendering of vibrotactile signals and sounds during tool-surface interactions. We develop a comprehensive end-to-end data-driven framework for the rendering of vibrotactile feedback and sounds. First, we capture contact acceleration signals and sound data from actual surfaces. Secondly, we construct the model using encoder-decoder networks. Finally, we render the vibrotactile feedback using an actuator connected to the stylus, while the sound is routed to the headphones. The proposed encoder-decoder networks consist of a stacked transformer and two one-dimensional convolutional layers. The stacked transformer takes into account both the local variability and the overall trend within a sequence by integrating the information from the lower layer, which has acquired relatively local details, and with the information from the upper layer that has captured global dependencies, respectively. To the best of our knowledge, this is the first attempt to apply transformer-based data-driven approach for modeling and rendering of vibrotactile feedback as well as sounds during tool-surface interactions. To exhibit the effectiveness of the proposed framework, we conducted numerical evaluation with six haptic textures samples. Furthermore, two different user studies (i.e., subjective similarity evaluation and virtual vibrotactile feedback guessing) are performed to establish the perceptual performance.

The paper is organized as follows. In section II, we provide a review of related works. Section III details the proposed framework for simultaneous modeling and rendering vibrotactile signals and sounds in tool-surface interactions. Section IV presents numerical evaluations and user studies. Lastly, in section V, we conclude our work.

## Related works

Tool-mediated haptic texture and sound feedback provide detailed information about the properties of textured surfaces, including roughness, hardness, and slipperiness^3,5,20^. Our approach is based on the data-driven methodology for modeling and rendering haptic textures in the form of vibrotactile feedback as well as sounds during interactions with stylus-based surfaces. In this section, we will review the previous works closely related to the proposed method.

### Texture modeling and rendering

Romano et al.^16^ designed a novel recording system to capture user interactions with textured surfaces, encompassing motion, force, and acceleration data. They subsequently proposed a technique to compress the recorded three-dimensional acceleration data into a single-dimensional signal. Utilizing linear predictive coding (LPC), they then transformed this unprocessed haptic data into a collection of frequency-domain texture models, which were subsequently rendered in real-time on a Wacom tablet. This rendering was facilitated using a stylus enhanced with compact voice coil actuators. Due to fluctuations in the user’s velocity and applied force during data acquisition, the acceleration signal is not stationary. To address this, Culbertson et al.^2^ employed the Auto-PARM algorithm to partition the signal into segments, enabling its representation as a piecewise autoregressive model. Subsequently, in^19^, the frequency-decomposed neural networks was presented for the purpose of modeling contact vibrations. Additionally, they developed a motorized apparatus to systematically capture contact accelerations. Nevertheless, in their cross-validation process, the initial output sequence was randomized, which could potentially result in varying synthesized vibrations. Abdulali et al.^18^ introduced the utilization of a Radial Basis Function Network (RBFN) to model anisotropic haptic textures. Subsequently, in a later work^17^, they presented a comprehensive visually guided data collection system designed to aid users in gathering acceleration signals with varying speed, force, and direction. In addition, they proposed a recursive constraint projection (RCP) algorithm for data segmentation, which also proves effective for processing streaming data. Hassan et al.^30^ presented the concept of Haptic Authoring, which involves the creation of novel virtual textures through the interpolation of pre-existing texture models, guided by their correlation with descriptive affective attributes. Vibrotactile signals are produced using Generative Adversarial Network (GAN) in^11^. Nai et al.^3^ proposed a rendering approach that involves identifying a representative waveform segment from the recorded acceleration signal. This technique utilizes a table to store these waveform segments, enabling bilinear interpolation across various speeds and normal forces of the tool-tip. In our previous work^4^, we formulated a deep spatio-temporal network (DSTN) aimed at data-driven modeling and rendering of haptic textures. Lu et al.^20^ developed a texture model generator based on Generative Adversarial Networks (GANs), enabling the creation of diverse texture models through Auto-Regressive functions. Their preference-driven interactive texture search approach employs an evolutionary strategy by incorporating feedback from the user’s preferred responses among a set of generated texture models.

### Sound modeling and rendering

Though various data-driven methods have been presented for rendering vibrotactile feedback, sound rendering has received relatively less attention. Lu et al.^5^ designed a data-driven modeling technique that produced naturalistic sounds through the utilization of audio signals obtained from unconstrained interactions between tools and surfaces. The approach involves segmenting captured audio, annotating each segment with its average velocity, and modeling individual segments using wavelet tree models through a moving window method. These models are created by applying fast wavelet transform and organizing windows into a tree structure. User velocity is used to select relevant tree models during rendering, and new sounds are generated operating breadth-first search and inverse wavelet transform methodology.

**Figure 1:**
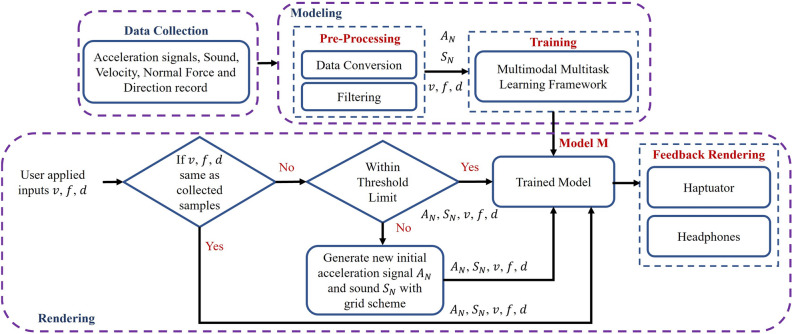
Proposed end-to-end data-driven system flow diagram for haptic textures and sounds rendering.

## Proposed framework

In this study, we developed an extensive end-to-end data-driven system that simultaneously models and generates vibrotactile feedback as well as sounds (see Fig. 1). Initially, we capture the vibrations occurring upon contact and record the resulting sounds produced when a physical tool interacts with a surface texture. Subsequently, we create a digital replica of this interaction using our state-of-the-art multimodal multitask deep learning model. Finally, we apply the recreated tactile sensations to a stylus pen using an actuator, while the synthesized virtual sound is directed to headphones for perception.

### Data collection

The majority of existing efforts in modeling and rendering gather data through two different methods: automated data collection using robotic systems^19^, and manual data collection involving users^2,4,5,17^. Similar to^17^, in this work, we adopt the manual data collection process with a simple user interface-based visual guidance. The interface assists the user in achieving the task using visual feedback for a specific force, velocity, and direction.

**Figure 2:**
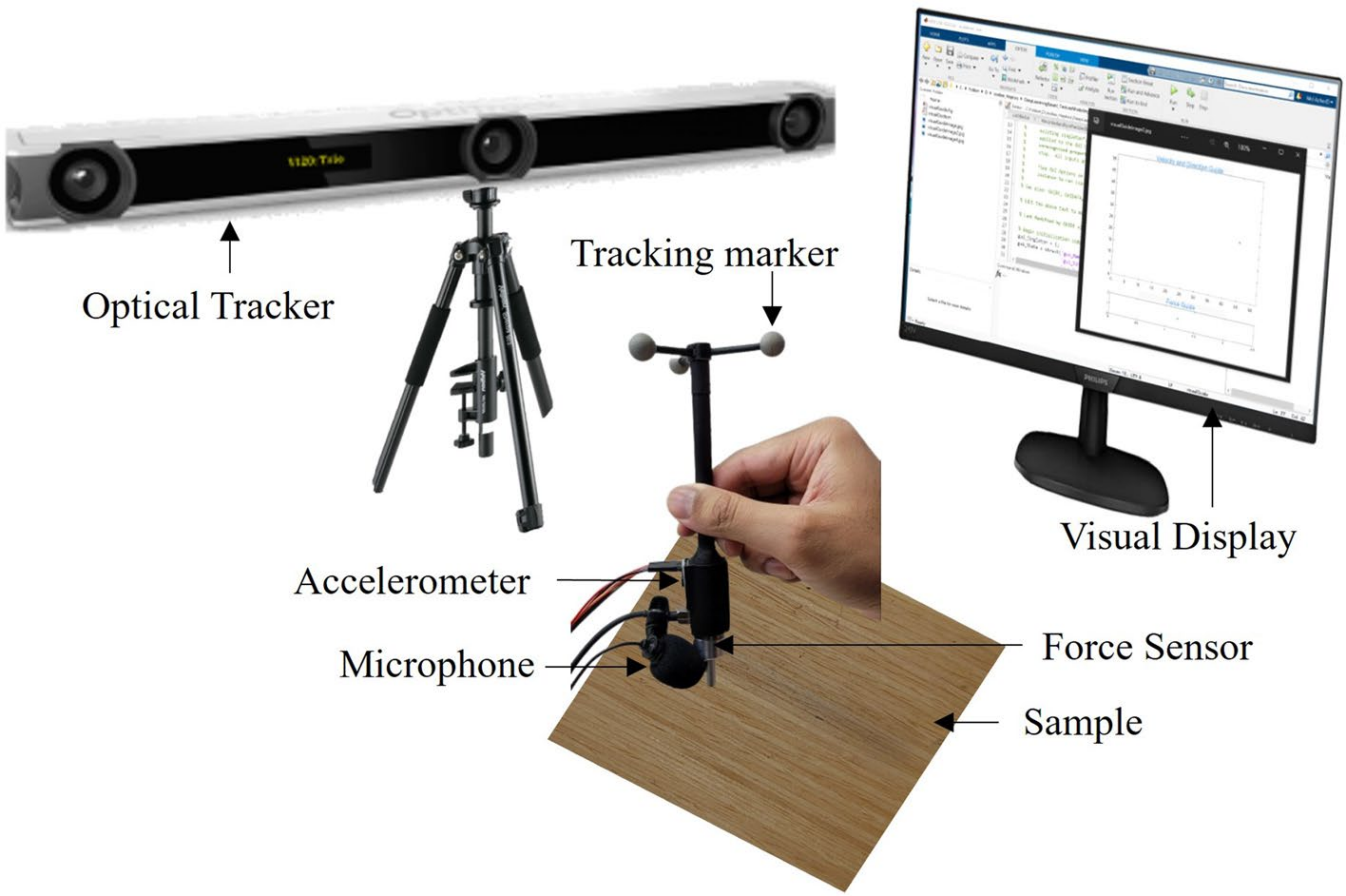
Data collection setup.

In this work, the system records the contact vibration as acceleration signals and sounds when a user moves the tool across a surface texture, considering specific parameters i.e., velocity, force, and direction (direction only for anisotropic textures). To achieve this, a custom-made 3D-printed haptic recording device is built. The device is equipped with an accelerometer (ADXL335), a microphone (Boya Lavalier Stereo Microphone), a force sensor (Nano17), and three snap-on reflective markers (for position tracking). We utilized the same material as described in^17^ for the tool-tip. To monitor the position of the tool-tip, an external position tracking system (Optitrack V120:Trio) is employed, operating at a sampling rate of 120 Hz. Simultaneously, an NI DAQ acquisition board gathers acceleration and force data at a rate of 2 kHz, while the microphone records sound at a frequency of 44.1 kHz. Although stereo sound is recorded, only the left channel sound is subsequently analyzed and processed, following a similar approach as^5^. The setup for data collection is illustrated in Fig. 2. The tool-tip’s velocity is determined based on the recorded position data. Filtering is applied to the force and velocity signals using a low-pass filter at a 25 Hz cutoff, while the high-pass filtering with a cutoff at 10 Hz is performed on the acceleration signal. Further data processing involves converting the three-axis acceleration signals into a single-axis signal using the DFT321 algorithm^2^. Subsequently, to increase the speed of processing of modeling, sounds are downsampled to 8 kHz. However, during rendering, the sound is upsampled back to 44.1 kHz. Importantly, downsampling without utilizing a filter does not alter the spectral density of the signal; as a result, the algorithm ensures that the modeling outcomes are equivalent to those obtained from the original signal^5^. Conversely, sinc-based resampling is used at the time of upsampling to offer higher accuracy and reduced artifacts.

**Figure 3:**
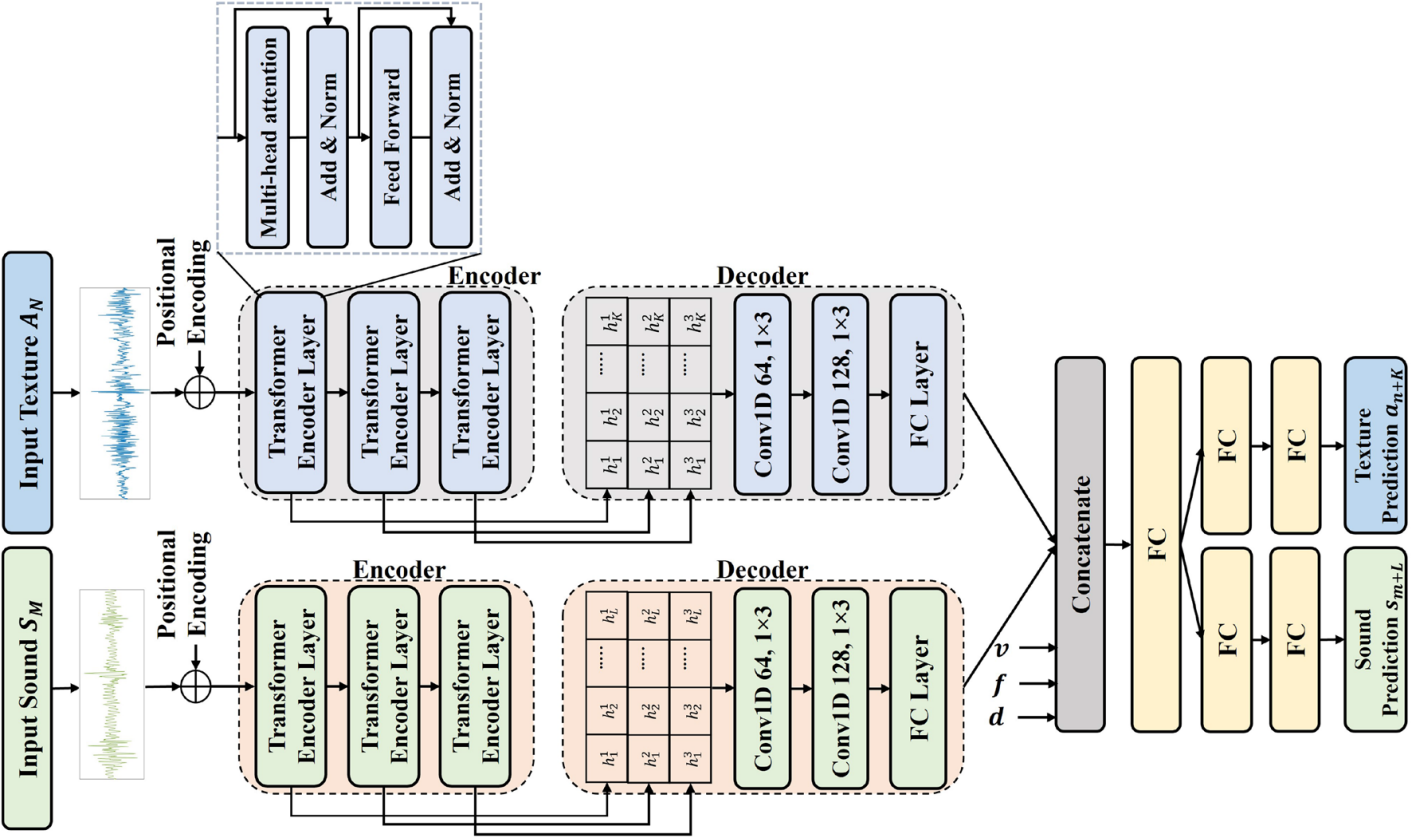
Proposed encoder-decoder network for simultaneous modeling of vibrotactile feedback and sounds.

### Vibrotactile signal and sound modeling

The acceleration signal and sound vary with the changes in user applied actions, i.e., velocity, and applied force as well as the scanning direction for anisotropic texture^2,4,5,17^. Therefore, the goal of this work is to design a multimodal network that maps the acceleration signals and sound with the user applied actions. In conventional approaches, the synthesis of acceleration signals^2,19^ and sounds^5^ was accomplished through the interpolation of neighboring models employing predetermined rules. However, deep learning models have the ability to autonomously acquire the interpolation rules by utilizing user-applied actions as input.

To this end, we formulate the problem as a multi-task learning based time-series data prediction task and present encoder-decoder based framework. This network is capable of simultaneously predicting both acceleration signals and sounds. Our proposed model, as illustrated in Fig. 3, comprises two encoder-decoder networks for acceleration signals and sounds, respectively. Each of these encoder-decoder networks incorporates a stacked transformer and two 1D convolutional layers, respectively. The design rationale behind this network is that traditional Convolutional Neural Networks (CNNs) emphasize local information, while Long Short-Term Memory networks (LSTMs) and Recurrent Neural Networks (RNNs) may struggle to capture long-range dependencies in sequences. Consequently, both models face the challenge of not adequately learning global trend from the given input sequence. This could be a primary factor contributing to performance degradation in high-frequency-based time-series forecasting tasks. Similarly, DSTN^5^ encounters the same issue as it combines 1D CNNs and LSTM networks. Therefore, a network capable of capturing the global trend of time-series data is required, which is achieved by the proposed model. The following subsections present further details.

#### Input of the model

The network takes scanning velocity v, applied force f, direction d, previous acceleration signal $A_N = \{a_n, a_{(n+1)}, a_{(n+2)}, ..., a_{(n+K-1)}\}$, and previous sound $S_M=\{s_m, s_{(m+1)}, s_{(m+2)}, ..., s_{(m+L-1)}\}$ as input, where *K* and *L* are the sizes of the input acceleration signal and input sound, respectively. Here, *n* and *m* denote the time points at which the acceleration signal and sound data begin. The network then synthesizes the acceleration data point $a_{(n+K)}$ and sound data point sound $s_{(m+L)}$ simultaneously. Note that, unlike previous works^4^, input signals are not segmented into partitions, as this could restrict the effective capture of global trend.

#### Encoder

The Transformer model was originally introduced for natural language processing (NLP), utilizing a self-attention mechanism to efficiently capture global dependencies within sequences. Beyond achieving success in machine translation, it has surpassed RNNs and CNNs in various NLP tasks. Recent developments have showcased its applicability in domains beyond NLP, including speech recognition and computer vision^25,26^. Notably, Transformer has been applied effectively to time-series analysis, particularly in forecasting tasks, as highlighted in studies such as^27,28^.

In this architecture, the encoder consists of an input layer, a positional encoding layer, and a set of three transformer layers. Initially, the input layer transforms the input time series data into a vector using a fully connected network, a crucial step to enable the subsequent multi-head attention mechanism. To incorporate temporal information, positional encoding using sine and cosine functions is employed by adding a positional encoding vector element-wise to the input vector. The resulting vector is then passed through three transformer layers, each of which consists of two sub-layers: a self-attention sub-layer and a fully connected feed-forward sub-layer. Following each sub-layer, there is a normalization layer applied. The input sequence goes through Transformer layers, generating a feature representation ${H}^i$ at each layer. To make use of the various feature representations learned across these layers, they are combined sequentially to create the final output representation ${H}^{(output)}$ of the encoder. More specifically,

1 $$\begin{aligned} {H}^1= & {} {TransformerLayer}^1(X_t) = \{{h}^1_1, {h}^1_2,..., {h}^1_q \} \end{aligned}$$

2 $$\begin{aligned} {H}^i= & {} {TransformerLayer}^i({H}^{i-1}) = \{{h}^i_1, {h}^i_2,..., {h}^i_q \}; where \; i \ge 2 \end{aligned}$$

3 $$\begin{aligned} {H}^{output}= & {} stack[{H}^1, {H}^2,..., {H}^p] \end{aligned}$$

Where, $X_t$ denotes the input signal, *q* is the size of input signal, *p* is the number of transformer encoder layers, ${h}^i_t$ $\in$ $\mathbb {R}^d$, and *d* refers to the dimension of the Transformer layer. In contrast to the conventional Transformer, which relies solely on the last output representation from the encoder, this study takes a different approach. It incorporates both local variations and the overall pattern within a sequence by merging insights from the lower layers, which capture more localized details, and upper layers, which capture global dependencies.

#### Decoder

The decoder takes the output representation from the encoder, incorporating the multi-level information it contains. To achieve this, a 1-dimensional convolutional neural network (1D CNN) is employed to merge the hierarchical information received from the encoder. The 1D CNN is composed of two convolutional layers and a fully connected layer. The convolutional layers apply 64 and 128 filters, respectively. The filter size is set to $1\times 3$. In contrast, the fully connected layer has 128 hidden nodes. The 1D CNN is effective in capturing time series data trends and cycles using fewer parameters compared to RNNs. Additionally, each filter in the CNN learns a consistent weight that remains unaffected by the passage of time.

#### Multitask learning

By leveraging information from multiple modalities (i.e., vibrotactile signal and sound branch), models can achieve better performance on tasks compared to using a single modality^22–24^ . This is because different modalities can provide complementary information that enriches the learning process. In our work, the features generated by both encoder-decoder networks are combined to extract shared characteristics from both modalities. In addition, multitask learning serves as a regularization technique, reducing the risk of overfitting to a specific task by forcing the model to learn more generalizable features^27^. The captured combined features, along with the scanning velocity (*v*), applied force (*f*), and direction (*d*), are then input into a fully connected layer (FC) with 256 hidden nodes. The scanning velocity (*v*), applied force (*f*), and direction (*d*) allow the model to automatically learn interpolation rules. These additional input parameters are normalized to ensure they are on a similar scale as the embeddings from the network. Then, the transformed inputs are concatenated with the embeddings before being passed to the fully connected layer. The next stage involves task-specific learning, where several FC layers are employed to capture features specific to the task. More specifically, in the case of acceleration signals, two FC layers with 128 and 64 hidden nodes are employed. Similarly, for the sound modality, two FC layers with 256 and 128 hidden nodes are utilized. To prevent overfitting, dropout is set to 0.4 after each FC layer, respectively. Finally, Sigmoid functions are utilized for the prediction tasks. In this study, the loss function utilized is the root-mean-square error (RMSE). To train the model, the ADAM optimization algorithm is used, employing a batch size of 32, a momentum of 0.9, and a learning rate set to 0.001. In the end, the network predicts 2000 data points per second for the vibrotactile signal branch and 8000 data points per second for the sound branch. During rendering, the sound is upsampled back to 44.1 kHz using sinc-based resampling.

**Figure 4:**
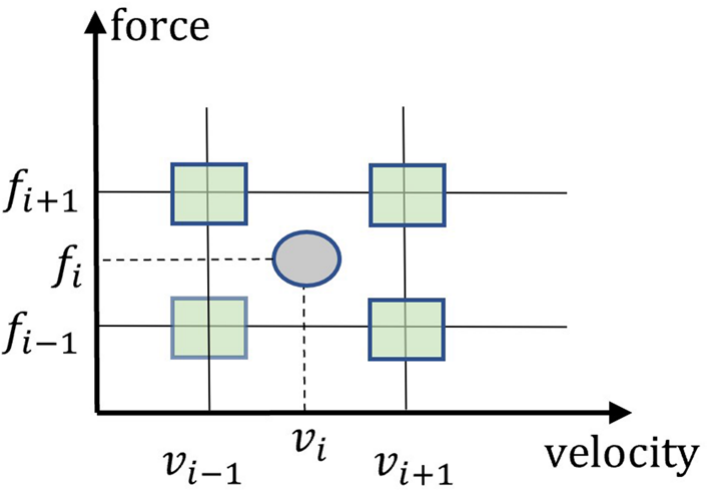
Velocity-force grid for cross-validation.

### Vibrotactile signal and sound rendering

In this study, we render vibrotactile signals to the voice-coil actuator (Haptuator MM1C; Tactile Labs) and virtual sound to headphones. This occurs when the stylus pen interacts with texture images displayed on the tablet (Microsoft Surface Pro 7) screen. The voice-coil actuator, connected to the stylus pen, provides tactile feedback to the user’s hand, and its operation is controlled through a USB soundcard and an amplifier. We utilize a Matlab function to simultaneously access the respective audioplayer objects (i.e., voice-coil actuator soundcard and headphones soundcard). Similar to our previous work^4^, we determine the stylus tip’s velocity by analyzing its position changes over time and measure the contact normal force through digital pressure readings from the stylus. The direction angle is calculated from two position vectors.

Note that if user applied actions do not match the collected velocity, force and direction, then there is no previous data (i.e., acceleration signals and sounds) at the beginning of contact. In that situation, motivated by^9,16,18^ we produce the initial acceleration sequence and sound data through employing the inverse distance weighting interpolation on the acceleration signal and sound of four neighboring conditions within the velocity and force grid (see Fig. 4) for isotropic haptic textures. Direction is irrelevant for isotropic haptic textures because they do not depend on a specific direction. More specifically, the initial acceleration sequence with size *K* and initial sound with size *L* are generated as follows.

4 $$\begin{aligned} A_N= & {} a(v_i,f_i) = w_{(p_{i-1},q_{i-1})} \times a(v_{i-1},f_{i-1}) + w_{(p_{i+1},q_{i-1})} \times a(v_{i+1},f_{i-1}) \nonumber \\{} & {} +w_{(p_{i-1},q_{i+1})} \times a(v_{i-1},f_{i+1}) + w_{(p_{i+1},q_{i+1})} \times a(v_{i+1},f_{i+1}) \end{aligned}$$

5 $$\begin{aligned} S_M= & {} s(v_i,f_i) = w_{(p_{i-1},q_{i-1})} \times s(v_{i-1},f_{i-1}) + w_{(p_{i+1},q_{i-1})} \times s(v_{i+1},f_{i-1}) \nonumber \\{} & {} + w_{(p_{i-1},q_{i+1})} \times s(v_{i-1},f_{i+1}) + w_{(p_{i+1},q_{i+1})} \times s(v_{i+1},f_{i+1}) \end{aligned}$$

6 $$\begin{aligned} w_{(p,q)}= & {} \frac{d_{(p,q)}^{-1}}{\sum _{t=1}^{N} d_{(p,q)_{t}}^{-1}} \end{aligned}$$

Where, *a* and *S* represents the acceleration signal and sound. *v* and *f* denote the velocity and force, respectively. *w* is the weight, which is computed by the inverse distance weighting method. $(i-1)$ and $(i+1)$ represent the previous and posterior positions for an arbitrary given position *i*. When dealing with anisotropic haptic textures, we also employ inverse distance weighting interpolation to calculate the initial acceleration sequence and sound data, which considers two neighboring directions. Additionally, in anisotropic haptic textures, we use a similar approach to isotropic textures to compute the initial acceleration sequence and sound data, taking velocity and force conditions into account.

Eventually, the generated initial acceleration sequence and sound data as well as scanning velocity, force, and direction are input into the proposed model to produce the following acceleration data and virtual sound, which are subsequently rendered to a voice-coil actuator at 2 kHz and headphones at 44.1 kHz, respectively. In this work, we have incorporated specific threshold values for user-applied actions (i.e., velocity, force, and direction). This addition serves a crucial purpose: it prevents the model from solely functioning as a grid-interpolation model, which could result in a continuous generation of initial data, ultimately could destroy the realism. Hence, our method examines if there is a substantial alteration in the user’s executed actions. In the event of such a change, the system proceeds to recalculate the initial acceleration sequence and sound data using the grid interpolation technique. Then, they are inputted into the model in conjunction with the user’s executed actions. If the user’s actions remain relatively consistent and fall within the defined threshold limit (i.e., Velocity threshold = $-0.5<v_{th}<0.5$ cm/s; Force threshold = $-0.1<f_{th}<0.1 N$; and Direction threshold = $-{15}^{\circ }<d_{th}<{15}^{\circ }$), the encoder-decoder networks proceed to generate subsequent acceleration signals and sounds using the previous acceleration signals ($A_N$) and previous sound data ($S_M$) with respective sizes of *K* and *L*. The threshold values were determined through a pilot user study. Note that the users who participated in the pilot study were not involved in the evaluation phase. This cycle continues until the user removes the tip of the stylus pen from the surface screen.

**Figure 5:**
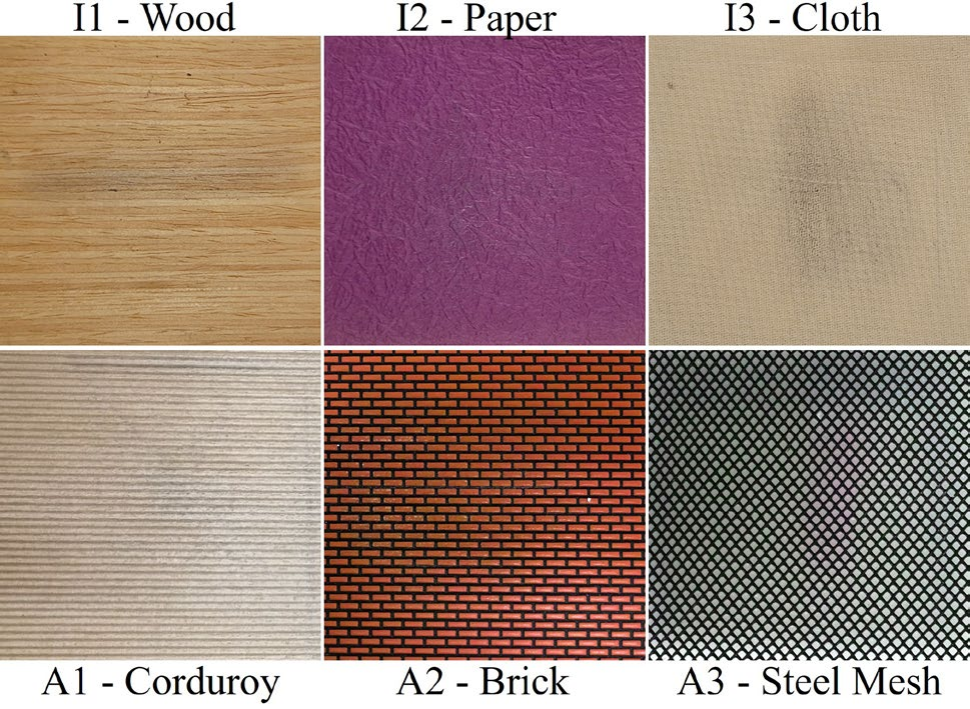
Six textured surface samples used for experiment.

## Performance evaluation

In this section, we provide an assessment of the proposed approach through experimentation. The evaluation is categorized into two subsections: one involves numerical assessment, and the other involves an evaluation based on user studies.

### Numerical evaluation

To assess the effectiveness of our approach, we gathered contact acceleration signals and sound data from a diverse set of haptic textures, encompassing both isotropic materials (i.e., wood, paper, and cloth) as well as anisotropic textures (i.e., corduroy, brick, and steel mesh) (see Fig. 5). Some of these materials are also used in^2,4,5,17,19,31^. Each acceleration and sound were 5 s long, encompassing a total of 10,000 samples recorded at a 2 kHz sampling rate for acceleration signals, and 220,500 samples recorded at a 44.1 kHz sampling rate for sound data. In our experimentation with both isotropic and anisotropic haptic textures, we employed five different velocities ($v = 10, 12, 14, 16,$ and 18 cm/s) and five normal forces ($f = 1.4, 1.7, 2.0, 2.3,$ and 2.6 *N*). Additionally, for anisotropic textures, we employed eight scanning directions ($d= {0}^{\circ }, {45}^{\circ }, {90}^{\circ }, {135}^{\circ }, {180}^{\circ }, {225}^{\circ }, {270}^{\circ }, {315}^{\circ }$). During the training phase of our proposed model, we specifically used data with velocities of 10, 14,  and 18 cm/s, normal forces of 1.4, 2.0,  and 2.6*N*, and scanning directions of ${0}^{\circ }, {45}^{\circ }, {90}^{\circ }, {135}^{\circ }$. The remaining data were reserved for cross-validation purposes. To assess the performance of our model, we utilized the relative spectral RMS error as a metric for comparing the synthesized sequences with the collected data for both acceleration signals and sound data.

**Figure 6:**
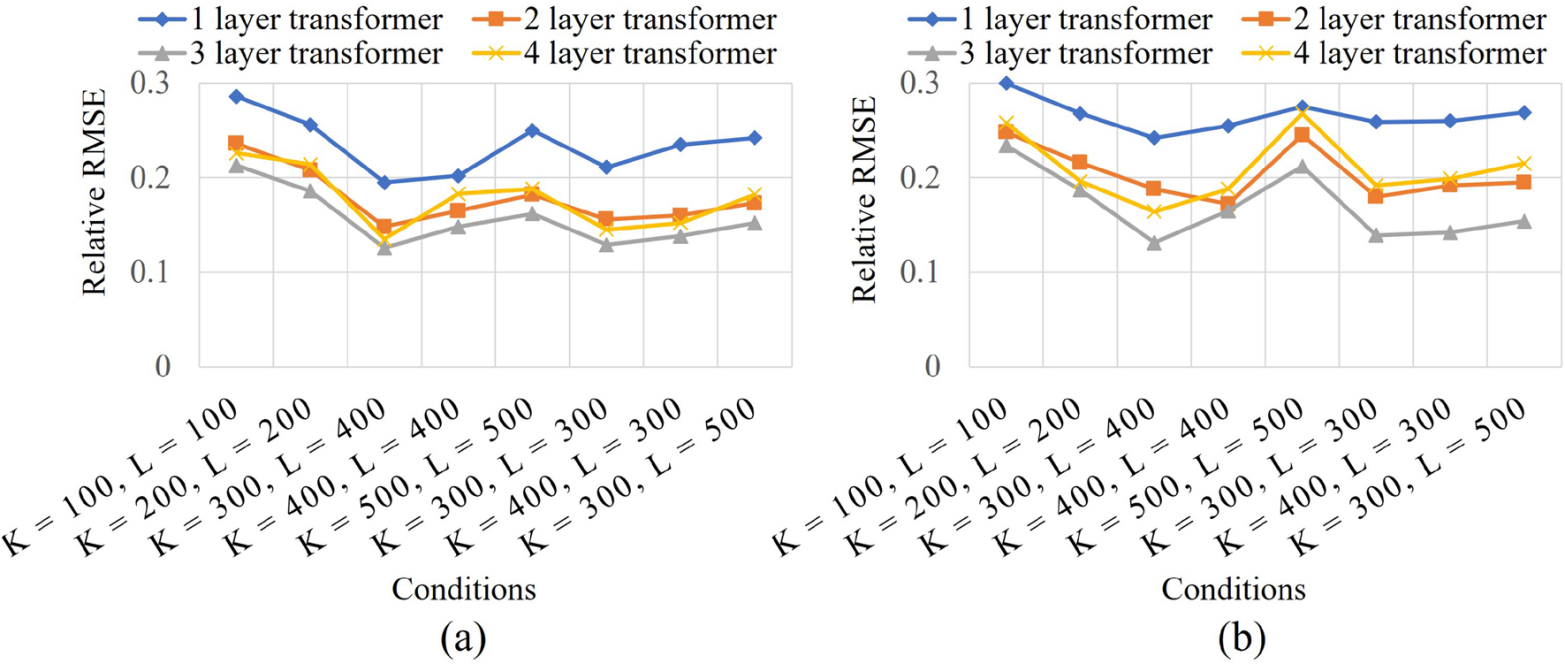
Experimental result with varying K and L for (**a**) Acceleration signals and (**b**) Sound data.

**Figure 7:**
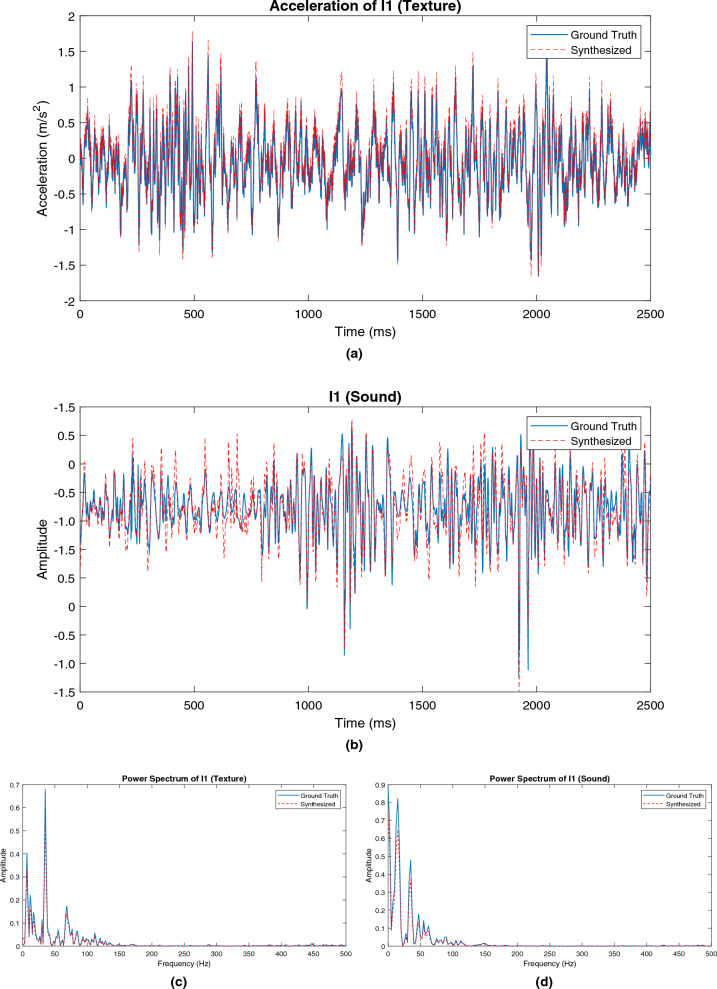
Measured and synthesized data: (**a**) Acceleration signal for wood (I1) with v = 16 cm/s and f = 2.3 N, (**b**) Sound data for wood (I1) with v = 16 cm/s and f = 2.3 N, (**c**) Power spectrum of acceleration signal from I1 and (**d**) Power spectrum of sound from I1.

Initially, we conducted an experiment aimed at identifying the ideal values for K and L in the context of both acceleration signals and sound data. Fig. 6 demonstrates the outcomes of this experiment across varying K and L sizes. The findings from this experiment revealed that our proposed approach achieves optimal performance when K is configured at 300, and L is set to 400. It was observed that as the size of K and L increased beyond this point, the error rate started to rise. This is because the proposed model struggled to effectively capture both local and global features in the data.

**Figure 8:**
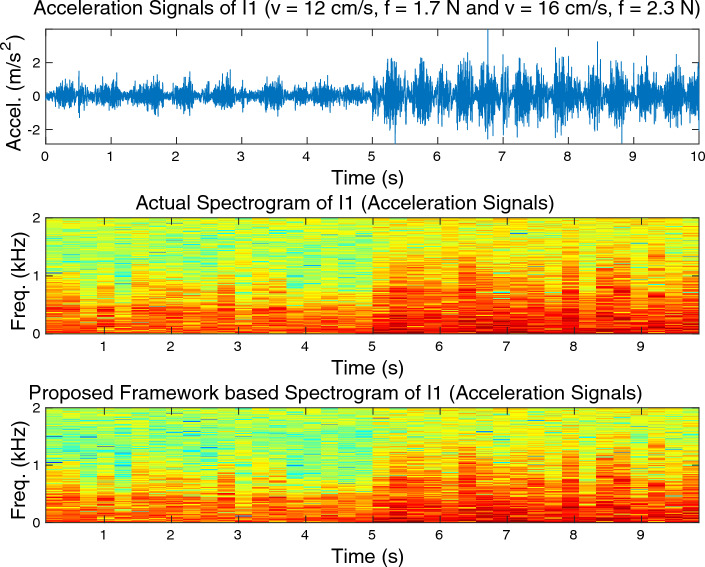
Spectrogram comparisons for the measured and synthesized cross-validation data with wood (I1) acceleration signal.

**Figure 9:**
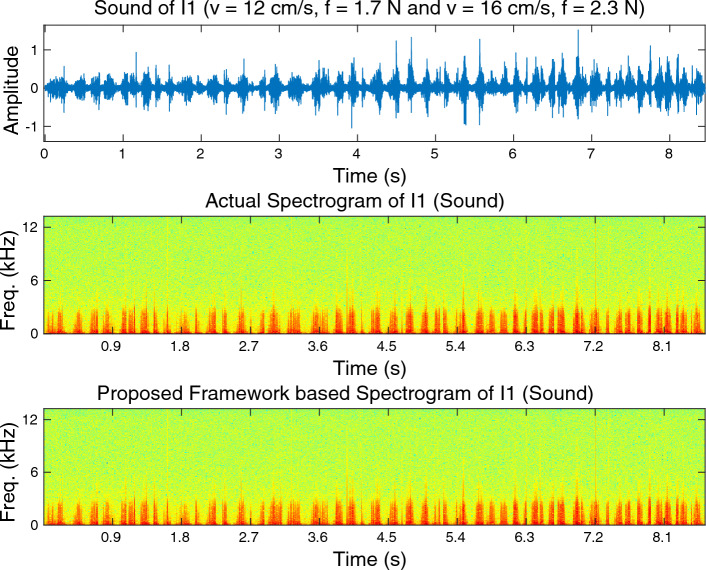
Spectrogram comparisons for the measured and synthesized cross-validation data with wood (I1) sound data.

Examples of measured and rendered signals are illustrated in Fig. 7 for both acceleration signal and sound data. Notably, the predicted trajectories closely align with the actual ground-truth data. This outcome is particularly encouraging since achieving such a high degree of accuracy in signal prediction tasks is typically quite challenging. Spectrogram comparisons for the measured and synthesized cross-validation data with wood (I1) acceleration signal and sound data are demonstrated in Figs. 8 and 9, respectively. A visual inspection of these plots validates that the proposed framework exhibits outstanding performance.

**Figure 10:**
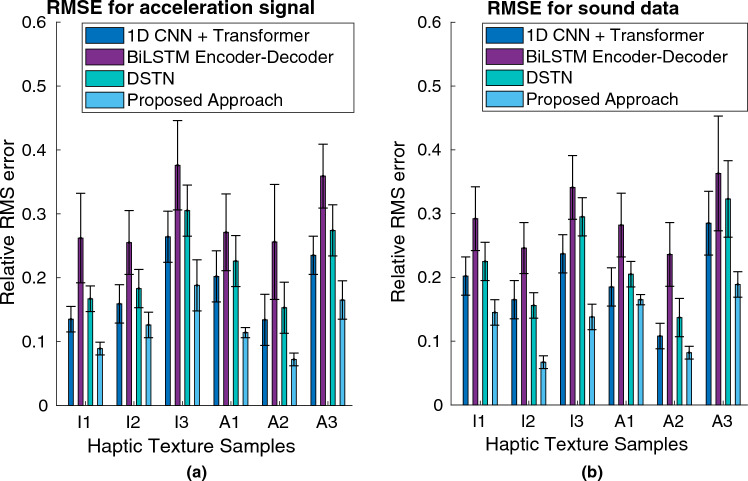
Simulation RMS error comparison with state-of-the-art deep models for (**a**) Acceleration signal and (**b**) Sound data. The error bars indicate standard deviation.

To present the impact of our encoder-decoder network, we compared the numerical error generated by our model with that of state-of-the-art deep networks, including 1D CNN + Transformer^33^, BiLSTM encoder-decoder^4^, and DSTN^4^. As depicted in Fig. 10, our approach consistently outperforms these alternatives in all cases, showing superior results. In this experiment, we observed that the brick texture sample (A2) demonstrated the lowest RMS error for acceleration signals, while the paper texture sample (I2) exhibited a lower RMS error for sound data. Conversely, the cloth texture sample (I3) displayed the highest RMS error for acceleration signals, and the steel mesh texture sample (A3) had the highest RMS error for sound data when employing our proposed approach.

**Figure 11:**
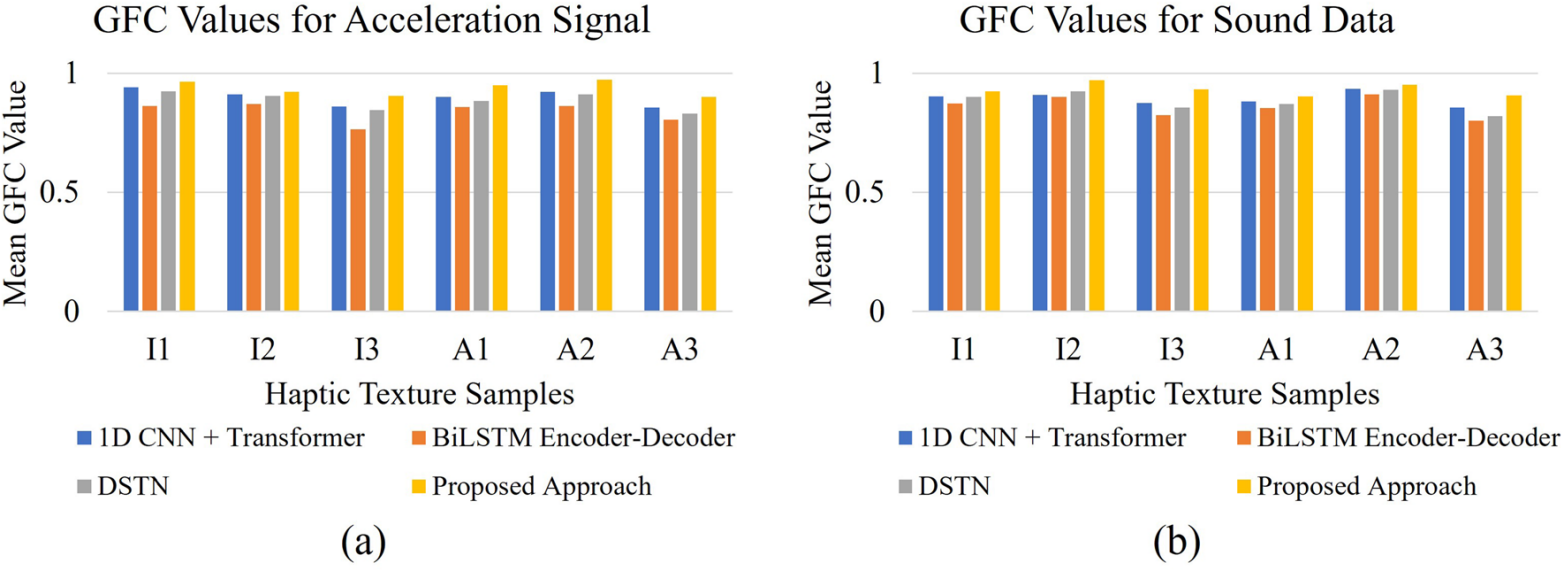
GFC value comparison with state-of-the-art deep models for (**a**) Acceleration signal and (**b**) Sound data.

To further evaluate the performance of the proposed approach for rendering vibrotactile signals and sound data, we employed a frequency spectral comparison method. This assessment involved using the Hernandez-Andres Goodness-of-Fit Criterion (GFC)^18^, which quantifies the error between the power spectrum of the rendered and measured acceleration signals. The GFC, derived from the Schwartz inequality, generates a value within a range of 0 to 1, where 1 signifies perfect reconstruction of the signals. GFC value comparison with state-of-the-art deep models for acceleration signal and sound data are demonstrated in Fig. 11(a) and Fig. 11(b), respectively. Notably, the results indicate that our proposed approach exhibited superior GFC values, with the brick texture sample (A2) demonstrating the highest GFC value for acceleration signals, and the paper texture sample (I2) yielding the highest GFC value for sound data. Conversely, the steel mesh texture sample (A3) exhibited the lowest GFC values for both acceleration signals and sound data when utilizing our approach. This experiment consistently demonstrates that across all employed texture samples, our approach outperforms existing state-of-the-art methods (e.g., 1D CNN + Transformer^33^, BiLSTM encoder-decoder^4^, and DSTN^4^) in terms of GFC values.

**Figure 12:**
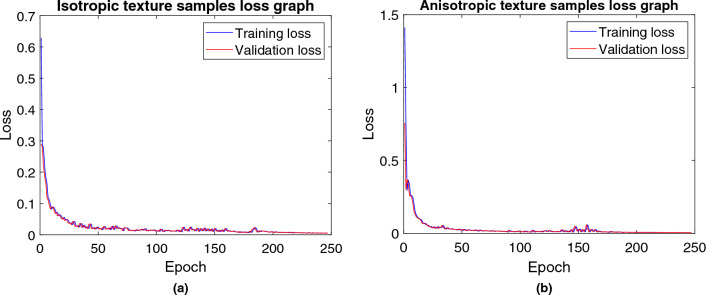
Training and validation loss of the proposed encoder-decoder network for (**a**) Isotropic texture samples and (**b**) Anisotropic texture samples.

We also calculated the training and validation loss of the proposed encoder-decoder network, as shown in Fig. 12. This experiment confirms that our model has not experienced overfitting, indicating its robustness and generalization capability. Lastly, we investigated the response time of the network during cross-validation. On average, our proposed model exhibited a response time of 8.4 milliseconds. Recent guidelines suggest that a response time for vibrotactile feedback should be between 5 and 50 milliseconds^32^. Therefore, our model’s response time falls well within the acceptable range.

### User study

In this study, two different user studies were carried out to assess the performance of the proposed rendering method. The first study involved subjective similarity evaluation, while the second study focused on vibrotactile feedback guessing. All methods were carried out in accordance with relevant guidelines and regulations as well as all experimental protocols were approved by the Institutional Ethical Committee at the authors’ institution.

**Figure 13:**
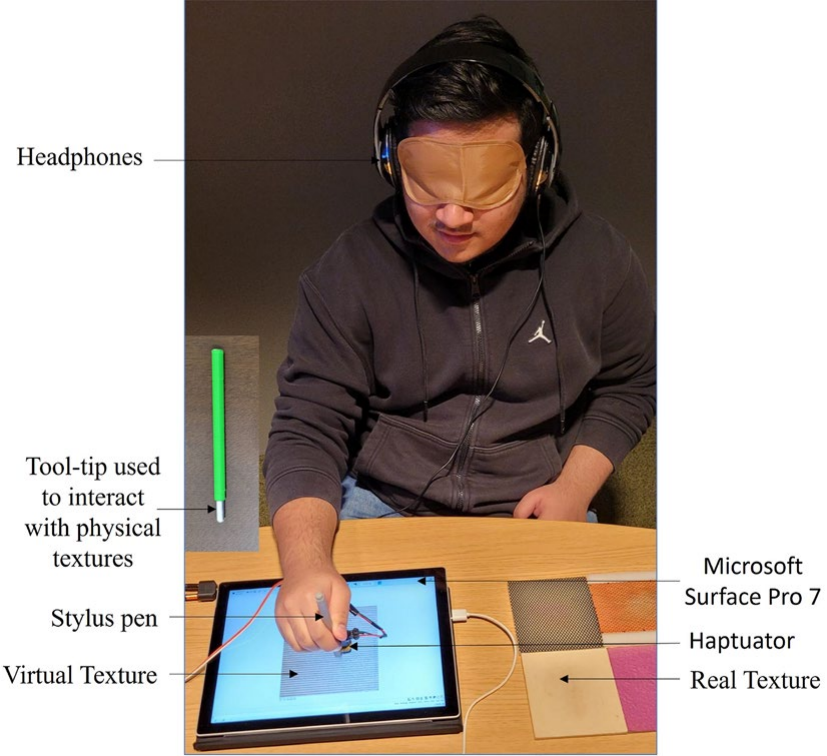
Rendering experiment.

#### Subjective similarity evaluation

In this section, we conducted a user study aimed at assessing subjective similarity. Participants were tasked with rating how similar the feedback rendered virtually was to the real feedback experienced from the physical surface interaction. The study involved a total of fifteen participants, comprising 9 males and 6 females (with an average age of 24.5 years old). None of the participants reported any disabilities, and they were provided with information about the experiment and requested to sign written consent. The informed consent is taken from all participants for both study participation and publication of identifying information or images. In appreciation of their participation, the participants received compensation of 30 AED ($\sim$ 8 USD). For this study, we utilized six haptic textures and employed four different rendering strategies: proposed virtual vibrotactile feedback, proposed virtual sound feedback, proposed virtual vibrotactile-sound feedback and DSTN based virtual vibrotactile-sound feedback. This setup led to a total of 30 stimuli conditions for the purpose of comparing similarity. Out of these, six were designated for real-virtual comparisons, and these comparisons were repeated for the four different rendering. Additionally, another six conditions served as reference points for comparing real-real pairs.

The experimental session was divided into two segments: a training session and a main session. In the training session, participants were familiarized with the texture surfaces and the corresponding feedback. They were informed that they would experience and hear vibration of real surface textures, as well as the rendered vibrotactile signals and sounds when they made contact with and stroked the surface in front of them. Additionally, participants were trained to rate the overall haptic similarity on a scale ranging from 0 to 100. During the main session, participants were seated in a chair and blindfolded. Each of the 30 pairs of stimuli was presented twice to every participant. In each trial, they were instructed to assess the similarity between the two sensations presented to them. On average, this entire experiment took approximately 75 minutes for each participant. The overall experimental setup can be seen in Fig. 13. Participants used the same tool-tip for subjective similarity evaluation to interact with physical textures, which were used during data capturing. Conversely, a stylus pen was used to interact with the virtual textures.

**Figure 14:**
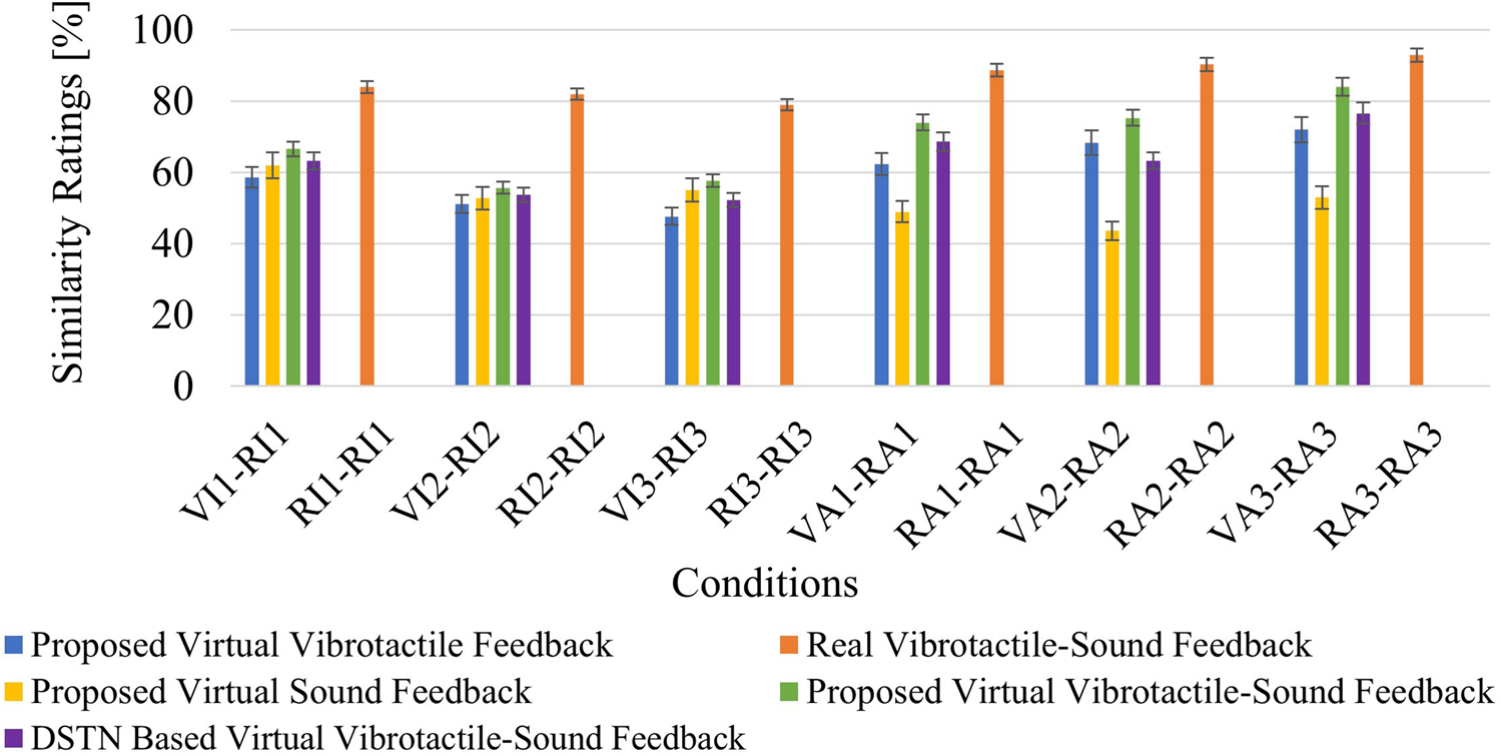
Mean similarity scores, with V denoting virtual and R representing real, while I stands for isotropic and A indicates anisotropic textures, followed by the respective texture number. Error bars on the graph represent the standard error of the mean.

The experimental findings, presented in Fig. 14, showcase average similarity ratings. The pair VA3-RA3 received the highest rating, with a score of 84% for the proposed virtual vibrotactile-sound feedback, while the lowest rating of 47.67% was observed for VI3-RI3 during the proposed virtual vibrotactile feedback. These results indicate that participants favored the combined virtual vibrotactile-sound feedback over individual feedback, particularly in the case of anisotropic textures. Additionally, during interviews, many participants mentioned their ability to clearly differentiate between virtual anisotropic textures and connect them with real textures when providing virtual vibrotactile feedback. However, in the case of solely virtual sound feedback, participants found it challenging to distinguish between virtual anisotropic textures, whereas sound feedback proved helpful for virtual isotropic textures. In this experiment, we also compared our approach with state-of-the-art DSTN^4^. The findings indicate a preference among participants for our proposed approach over the existing method. After confirming that the assumptions of ANOVA were met, we conducted a repeated measures ANOVA, which revealed a significant difference among the various rendering approaches in terms of the scores ($F = 16.92, p = 2.69 \times {10}^{-10}, {\eta }^{2} = 0.12$). Bonferroni-Holm pair-wise post-hoc analysis tells that except proposed virtual sound feedback vs. DSTN based virtual vibrotactile-sound feedback ($p = 0.5802$), all other methods are significantly different (i.e., proposed virtual vibrotactile feedback vs. proposed virtual sound feedback $p = 0.0077$, proposed virtual vibrotactile-sound feedback vs. proposed virtual sound feedback $p = 8.33 \times 10^{-4}$, proposed virtual vibrotactile-sound feedback vs. proposed virtual vibrotactile feedback $p = 1.45 \times 10^{-11}$, DSTN based virtual vibrotactile-sound feedback vs. proposed virtual vibrotactile feedback $p = 4.68 \times 10^{-5}$, and proposed virtual vibrotactile-sound feedback vs. DSTN based virtual vibrotactile-sound feedback $p = 0.0089$).

#### Virtual vibrotactile feedback and sound guessing

This section outlines the user study conducted to evaluate the virtual vibrotactile feedback and sound guessing performance. Participants were presented three different feedbacks (i.e., virtual vibrotactile feedback, virtual sound feedback, and virtual vibrotactile-sound feedback together) of a texture surface and asked to find the best-matching real surface. For each feedback, participant compares the virtual feedback and real surface feedback, if a participant properly matches the feedback, then it is counted as 1, otherwise 0. At the end these values are used to produce the confusion matrices. A total of 15 participants, comprising 11 males and 4 females with an average age of 23.5 years, participated in this study. These participants are different from similarity measure user study to ensure that there were no learning effects from a previous similarity measure user study. All participants confirmed that they did not have any disabilities and were informed about the details of the experiment, after which they were asked to sign a written consent form. The informed consent is taken from all participants for both study participation and publication of identifying information or images. As a token of appreciation for their involvement, participants received compensation amounting to 30 AED ($\sim$ 8 USD).

**Figure 15:**
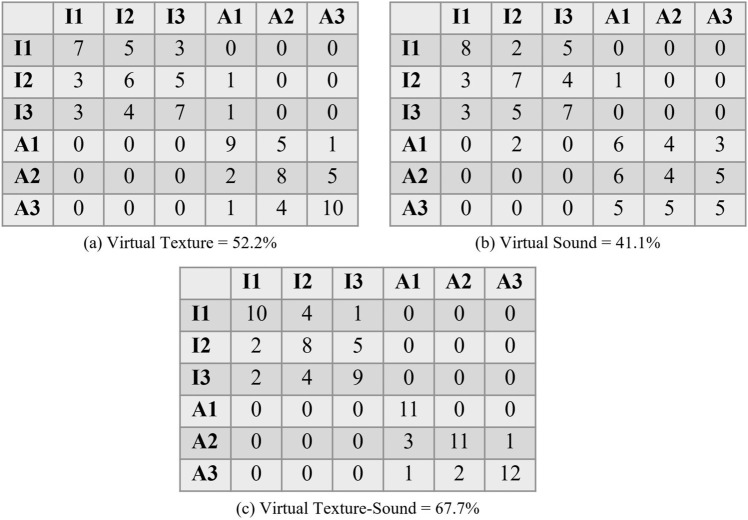
Confusion matrices presenting the virtual feedback guessing outcomes made by 15 participants for each of the three rendering strategies: (**a**) virtual vibrotactile feedback, (**b**) virtual sound feedback and (**c**) virtual vibrotactile-sound feedback.

Six different haptic textures were utilized for comparison in the study. The experimental session consisted of two parts: a training session and a main session. During the training session, participants were introduced to real texture surfaces and the associated feedback. They were informed that they would both feel and hear vibrations from these real surface textures, as well as perceive both virtual tactile vibrations and auditory sensations when they touched and stroked the surface in front of them. In the main session, participants were seated in a chair and had their vision obstructed by wearing an eye mask. Note that, during the virtual vibrotactile feedback, participants wore headphones playing white noise to block auditory distractions, while in other cases, virtual sounds were generated. The sequence of virtual feedback for each strategy was randomly determined. The participants’ task was to correctly identify the corresponding real texture for each virtual feedback.

In the study on virtual vibrotactile feedback and sound recognition, the results indicated recognition rates of 52.2% for virtual vibrotactile feedback, 41.1% for virtual sound rendering, and a notably higher rate of 67.7% when both virtual vibrotactile and sound feedback were combined, as depicted in Fig. 15. The experiment validates that the integration of both types of feedback enhances realism and, consequently, participants’ guessing accuracy. From the analysis of the confusion matrices, it is apparent that there was some confusion between paper and cloth when virtual vibrotactile feedback was employed. Similarly, participants had difficulty distinguishing between corduroy, brick, and steel mesh when sound feedback was used. On the other hand, when participants received virtual vibrotactile-sound feedback together, they were able to clearly differentiate between isotropic and anisotropic textures.

## Conclusion

This paper introduces a novel multimodal multitask deep learning framework, which enables the rendering of vibrotactile feedback and sound simultaneously, enhancing the realism and naturalism of virtual experiences. Encoder-decoder networks comprises of stacked transformer and two one-dimensional convolutional layers are utilized for modeling the contact acceleration signals and sound data, respectively. Finally, conducted user studies demonstrated the effectiveness of proposed framework. The results highlight the advantage of combining virtual vibrotactile and sound feedback over individual modalities. In the future, we will use data augmentation to enhance the model’s robustness and generalization capabilities. In addition, we will perform experiments with larger and more diverse group of participants.

## Data Availability

The dataset used during the current study are available in https://azher006.wixsite.com/home/projects.

## References

[1] Chan, S., Tymms, C., & Colonnese, N. Hasti: Haptic and audio synthesis for texture interactions. In *Proceedings of the IEEE world haptics conference (WHC), Montreal, QC, Canada*, pp. 733–738, (2021). 10.1109/WHC49131.2021.9517177.

[2] Culbertson H, Unwin J, Kuchenbecker KJ (2014). Modeling and rendering realistic textures from unconstrained tool-surface interactions. IEEE Trans. Haptics.

[3] Nai W, Liu J, Sun C, Wang Q, Liu G, Sun X (2021). Vibrotactile feedback rendering of patterned textures using a waveform segment table method. IEEE Trans. Haptics.

[4] Joolee JB, Jeon S (2022). Data-driven haptic texture modeling and rendering based on deep spatio-temporal networks. IEEE Trans. Haptics.

[5] Lu S, Chen Y, Culbertson H (2020). Towards multisensory perception: Modeling and rendering sounds of tool-surface interactions. IEEE Trans. Haptics.

[6] Siira, J. & Pai, D. K. Haptic texturing-a stochastic approach. In *Proceedings of IEEE international conference on robotics and automation, Minneapolis, MN, USA* **1**, 557–562. 10.1109/ROBOT.1996.503834 (1996).

[7] Fritz, J.P., & Barner, K.E. Stochastic models for haptic texture. In *Telemanipulator and Telepresence Technologies III.* Vol. 2901, pp. 34–44, SPIE, (1996).

[8] McDonald, C.G., & Kuchenbecker, K.J.. Dynamic simulation of tool-mediated texture interaction, Proceedings of IEEE world haptics conference (WHC), Daejeon, Korea (South), pp. 307–312, (2013). 10.1109/WHC.2013.6548426.

[9] Zhu, X., & Wyse, L. Sound texture modeling and time-frequency LPC. In *Proceedings of the 7th international conference on digital audio effects (DAFX-04)*, (2004).

[10] Zheng, C. & James, D. L. Toward high-quality modal contact sound. In *Proceedings of the ACM SIGGRAPH, association for computing machinery, New York, NY, USA, Article* **38**, 1–12. 10.1145/1964921.1964933 (2011).

[11] Ujitoko Y, Ban Y, Hirota K (2020). GAN-based fine-tuning of vibrotactile signals to render material surfaces. IEEE Access.

[12] Ren Z, Yeh H, Lin MC (2013). Example-guided physically based modal sound synthesis. ACM Trans. Graph..

[13] Okamura, A.M., Dennerlein, J.T., & Howe, R.D. Vibration feedback models for virtual environments. In *Proceedings of the IEEE international conference on robotics and automation*, (1998).

[14] Ju, Y., Zheng, D., Hynds, D., Chernyshov, G., Kunze, K., & Minamizawa, K. Haptic empathy: Conveying emotional meaning through vibrotactile feedback. In *Proceedings of extended abstracts of the 2021 CHI conference on human factors in computing systems*, pp. 1–7, (2021).

[15] Minamizawa, K., Kakehi, Y., Nakatani, M., Mihara, S. & Tachi, S. TECHTILE toolkit: a prototyping tool for design and education of haptic media. In *Proceedings of the virtual reality international conference* 1–2 (2012).

[16] Romano JM, Kuchenbecker KJ (2012). Creating realistic virtual textures from contact acceleration data. IEEE Trans. Haptics.

[17] Abdulali A, Atadjanov IR, Jeon S (2020). Visually guided acquisition of contact dynamics and case study in data-driven haptic texture modeling. IEEE Trans. Haptics.

[18] Abdulali, A., & Jeon, S. Data-driven modeling of anisotropic haptic textures: Data segmentation and interpolation. In *Proceedings of the international conference on human haptic sensing and touch enabled computer applications*, pp. 228–239, (2016).

[19] Shin, S., Osgouei, R.H., Kim, K., & Choi, S. Data-driven modeling of isotropic haptic textures using frequency-decomposed neural networks. In *Proceedings of the IEEE world haptics conference (WHC)*, pp. 131–138, (2015).

[20] Lu S, Zheng M, Fontaine MC, Nikolaidis S, Culbertson H (2022). Preference-driven texture modeling through interactive generation and search. IEEE Trans. Haptics.

[21] Sterling A, Lin MC (2016). Integrated multimodal interaction using texture representations. Comput. Graph..

[22] El-Sappagh S, Abuhmed T, Islam SMR, Kwak KS (2020). Multimodal multitask deep learning model for Alzheimer’s disease progression detection based on time series data. Neurocomputing.

[23] Sawhney, R., Mathur, P., Mangal, A., Khanna, P., Shah, R.R., & Zimmermann, R. Multimodal multi-task financial risk forecasting. In *Proceedings of the 28th acm international conference on multimedia. association for computing machinery, New York, NY, USA*, pp. 456–465, (2020). 10.1145/3394171.3413752.

[24] Hong C, Yu J, Zhang J, Jin X, Lee KH (2019). Multimodal face-pose estimation with multitask manifold deep learning. IEEE Trans. Industr. Inf..

[25] Kim S, Gholami A, Shaw A, Lee N, Mangalam K, Malik J, Mahoney MW, Keutzer K (2022). Squeezeformer: An efficient transformer for automatic speech recognition. Adv. Neural Inform. Process. Syst..

[26] Liu J, Guo J, Xu D (2022). GeometryMotion-transformer: An end-to-end framework for 3D action recognition. IEEE Trans. Multimed..

[27] Li Z, Zhang X, Dong Z (2022). TSF-transformer: A time series forecasting model for exhaust gas emission using transformer. Appl. Intell..

[28] Thwal, C.M., Tun, Y.L., Kim, K., Park, S.-B., & Hong, C.S. Transformers with attentive federated aggregation for time series stock forecasting. In *Proceedings of the international conference on information networking (ICOIN)*, Bangkok, Thailand, pp. 499–504, (2023). 10.1109/ICOIN56518.2023.10048928.

[29] Osgouei RH, Kim JR, Choi S (2020). Data-driven texture modeling and rendering on electrovibration display. IEEE Trans. Haptics.

[30] Hassan W, Abdulali A, Jeon S (2020). Authoring new haptic textures based on interpolation of real textures in affective space. IEEE Trans. Industr. Electron..

[31] Coe P, Evreinov G, Raisamo R (2023). The impact of different overlay materials on the tactile detection of virtual straight lines. Multimodal Technol. Interaction.

[32] Kaaresoja Topi, Brewster Stephen, Lantz Vuokko (2014). Towards the temporally perfect virtual button: Touch-feedback simultaneity and perceived quality in mobile touchscreen press interactions. ACM Trans. Appl. Percept..

[33] Chen Yuhang, Yang Shuchen, Li Huan, Wang Lirong, Wang Bidou (2023). Prediction of sleep apnea events using a CNN-transformer network and contactless breathing vibration signals. Bioengineering.

